# SWOT analysis and revelation in traditional Chinese medicine internationalization

**DOI:** 10.1186/s13020-018-0165-1

**Published:** 2018-01-25

**Authors:** Haitao Tang, Wenlong Huang, Jimei Ma, Li Liu

**Affiliations:** 1 China Pharmaceutical University, Longmian Road 639, Nanjing, 211198 China; 2 Jiangsu Suzhong Pharmaceutical Group Co., Ltd., No. 1, Suzhong Road, Jiangyan District, Taizhou, 225500 Jiangsu China

**Keywords:** TCM, Internationalization, SWOT analysis

## Abstract

Traditional Chinese medicine (TCM) is currently the best-preserved and most influential traditional medical system with the largest number of users worldwide. In recent years, the trend of TCM adoption has increased greatly, but the process of TCM internationalization has suffered from a series of setbacks for both internal and external reasons. Thus, the process of TCM internationalization faces formidable challenges, although it also has favourable opportunities. Using SWOT analysis, this paper investigates the strengths, weaknesses, opportunities and threats for TCM. These findings can serve as references for TCM enterprises with global ambitions.

## Background

At present, the pace of the expansion of traditional Chinese medicine (TCM) into the overseas market continues to accelerate. Both the Chinese government and Chinese medicine enterprises are making efforts to promote TCM as it moves into the overseas market. However, particular issues associated with TCM, such as its complex composition, various effects of its actions, insufficient systematic studies during its internationalization, etc., hinder the adoption of specific TCM drugs in the internationally recognised pharmaceutical market. In this study, we use the SWOT method to analyse the current situation of TCM internationalization, and the analysis is divided into four aspects.

## Strength analysis of the internationalization of traditional Chinese medicine

### Strengths of traditional Chinese medicine in theory and in treating disease

There are many strengths of TCM in the treatment of diseases which are not found in Western medicine (Table 1). First, TCM and Western medicine are two different medical theoretical systems with different modes and substantial differences in such aspects as theoretical foundations, methods of thinking, as well as diagnostic and treatment approaches. In the latter, the treatments are partial; in the former, symptoms and root causes are treated simultaneously. TCM has a unique theory as well as diagnostic and treatment techniques and methods. Compared with modern Western medicine, which focuses on detailed molecular targets, TCM thought takes an overall approach and pays attention to syndrome differentiation based on an overall analysis of the illness and the patient’s condition. TCM adopts multiple levels (dimensions) and a multi-targeted method to make overall adjustments and restore the human body to achieve the goal of treating both the manifestation of the disease and its cause.

**Table 1:** Comparison of TCM and Western medicine in the treatment of diseases

Subjects	Characteristics
TCM	Based on the traditional Chinese medicine theory Symptoms and root causes are treated simultaneously Only less adverse side effects TCM works slowly and requires a long course of treatment
Western medicine	Based on the Western medicine theory The treatments are partial Produce some adverse side effects Western medicine works quickly

Second, TCM can be used to treat various diseases and especially has advantages for treating incurable diseases and chronic diseases [1, 2]. Berberine exhibits beneficial anti-inflammation effects for the inflammatory bowel diseases, it also differentially modulates the activities of ERK, p38 MARK, and JNK to suppress Th17 and Th1 T cell differentiation indicating that it could be a potential therapeutic drug to treat type 1 diabetes mellitus (DM) [3]. Even for a disease without an obvious clinical manifestation according to Western medicine principle, TCM treatment can use its theoretical advantages of syndrome differentiation and effective timing to relieve the disease and make up for the deficiencies of modern Western medicine [4, 5]. For example, in management of metabolic syndrome, TCM is an excellent representative in alternative and complementary medicines with a complete theory system and substantial herb remedies. Ginseng, rhizoma coptidis (berberine, the major active compound) and bitter melon were discussed for their potential activities in the treatment of metabolic syndrome [6–11]. Recently, a search of active ingredient(s) from some commonly used TCM has revealed a wide variety of compounds that are biologically active with therapeutic potentials. About 62% of the 240 species were found to contain chemical compounds with pharmacological procies were found for the treatment of at least one disease and 53% of them for two or more diseases [12]. Virtual mapping between databases of Chinese herbal ingredients and molecular targets of diseases is likely to offer a new avenue for drug discovery [13].

Third, some new TCM drugs are being successfully developed based on many years of clinical practice; these drugs can generally have precise effects and have only less adverse side effects [14]. Only minimal adverse effects were reported for Chinese medicines used in treating type 2 DM indicating certain advantages in the prevention of diabetes and delay of its complications [15].

At the same time, combined traditional Chinese and Western medicine has good effect in the treatment for patients which is beneficial to improve patients’ quality of life [16]. For example, TCM in combination with insulin exhibited better clinical effect in the treatment of gestational diabetes [17].

### Strengths in international demand for TCM

In present-day society, the concept of healthy living has undergone tremendous changes, and people are increasingly pursuing improved health and quality of life. The unique effects of TCM are not only valued by domestic intellectuals but have also attracted the attention of the international community. Correspondingly, the demand for natural botanical medicine is also increasing.

Compared with the research and development (R&D) of TCM, there are some problems in R&D of chemical medicine. First, the process of chemical drug discovery is long and arduous that it begins from the search of a potential candidate to the development of a marketable drug. The course could be as long as more than a decade [18]. Second, the R&D cost for a new drug can be, in average, more than 800 million USD in the United States (US) [19]. Third, the development of new chemical drugs remains very a low rate of success. Among thousands chemical compounds only a few candidates could reach their first markets as new drugs in recent years. Finally, synthetic chemical drugs are often associated with undesirable side effects in patients. It is now clear that the need of therapeutic intervention in many clinical conditions cannot be satisfactorily met by synthetic chemical drugs. Since the research and development of new chemical drugs remain time-consuming, capital-intensive, safety issues, and undesired side effects, much effort has been put in the search for alternative routes for drug discovery in China [20]. TCM has a long history of use, with extensive literature and clinical applications covering thousands of years. Such as berberine, an active ingredient from Coptis chinensis Franch, is widely used for the treatment of infectious diseases in China [21]. As TCM has the advantages of treatment of special diseases safety, and so on, there are many countries and regions begin to study it. At present, more than 150 countries and regions [22] have established natural botanical institutions, and pharmaceutical companies are increasingly focusing on research and development of botanicals, paying attention to the construction of traditional botanical studies and development teams, and focusing on the search for effective natural medicines to replace chemical treatments. Furthermore, there are over 1300 medicinal plants used in Europe [23]. As safe and healthy treatments are associated with a return to nature, TCM can make up for the shortcomings of Western medicine in many areas. We can find the solutions to different kinds of diseases that are hard to cure by using the innovations and developments of TCM. TCM displays a distinctive curative effect for different diseases that are hard to cure and for technological difficulties that are recognized worldwide, such as tumours [24], chronic liver disease [25] and chronic kidney disease [26].

### Strengths of natural medicine resources

China has rich natural medicine resources, with the world’s largest treasure of Chinese herbal medicine, and has a unique advantage in nurturing and developing the TCM industry. According to statistical studies, there are 12,807 TCM resources in China, including 11,146 medicinal plants, 1581 medicinal animals, and 80 medicinal minerals. At present, there are more than 600 commonly used Chinese medicinal materials, in which near 400 plant and animal species are artificially cultured. In addition, a statistical analysis of 320 commonly used plant medicinal materials indicates that the total resources storage has reached approximately 8.5 million tons [27].

At present, domestic cultivation is a widely used and generally accepted practice in China. Cultivation provides the opportunity to use new techniques to solve problems encountered in the production of medicinal plants, such as toxic components, pesticide contamination, low contents of active ingredients, and the misidentification of botanical origin. Cultivation under controlled growth conditions can improve the yields of bioactive components and obtain improved yields of target products [28].

### Strengths in the scale and power of TCM enterprises

China has a number of large-scale TCM enterprises with strong scientific research abilities. Chinese TCM enterprises are strengthening their research and development abilities while continuing to conduct studies of listed products and move forward with adaptions for the international market. At the same time, drug regulatory authorities continue to promote Chinese production and quality management systems in connection with international standards and to improve the good manufacturing practice (GMP) management level. Chinese TCM enterprises should achieve the GMP requirements and make efforts to connect to the international production management system. They have increased their investment in technological transformation so that the TCM production quality management level has reached a historical new height through the improvement of the production environment and conditions as well as the promotion of staff quality and production quality management.

In addition, in recent years, upstream and downstream enterprises and industries related to TCM, such as Chinese herbal planting, research and development; the manufacture and marketing of pharmaceutical equipment; TCM transportation; and many other supporting industries, have continued to grow and develop through the promotion and rapidly expanding development of the TCM industry. Thus, the development of the TCM industry can also advance the development of supporting industrial chains, forming a favourable interaction.

## Weakness analysis of the internationalization of TCM

We have analyzed the strengths of the internationalization of TCM, but we should also realize that many unfavourable factors are also related to the internationalization of TCM.

### Cultural diversity

Chinese medicine theory has many ancient Chinese-based terms that cannot be expressed in objective and modern scientific language and evidence, so that many Chinese medicine terms, such as yin-yang and the five elements, assistant and guide and other traditional theories and terms, are not understood and accepted by the international community [29]. TCM is a medical system developed on the basis of Taoist philosophy. The theory of TCM was first documented in an ancient Chinese book, Huangdi Neijing (Yellow Emperor’s Inner classic). The book proposes that the human body contains Yin and Yang. A disease is a consequence of disbalance of Yin and Yang. Qi (air) and blood serve as mediators in communication between Yin and Yang. The primary aim in the treatment of illness is to restore the balance, and replenish Qi or blood. Herbal medicines, acupuncture, and massage are often used to restore the balance in the clinical practice in TCM [2, 30].

Eastern and Western cultures are different not only in language systems but also in values, ways of thinking, etc. Chinese medicine and pharmacy are a huge, complex system of long-term experience based practice, emphasizing an overall view that is totally different from the perspective of Western medicine and pharmacy. In the TCM theory, diabetes is considered a result of Yin deficiency with dryness-heat. The treatment of diabetes should be focused on replenishing Yin (fluid) and evacuating fire (heat) from the body [31]. However, for Western medicine, such as Metformin is commonly used in the treatment of diabetes. It acts primarily by decreasing hepatic glucose output, largely by inhibiting gluconeogenesis [32].

Chinese medicine is based on a holistic approach, while Western medicine is based on a reductionistic approach. For example, in the treatment of cancer, Western medicine often take the tumor mass reduction as the ultimate goal, regardless of normal cells; while the TCM in addition to tumor inhibition, but also intend to alleviate the symptoms, strengthen the body resistance, improve the quality of life, prolong the survival time of cancer patients for the therapeutic purpose [32, 33].

### Weakness in quality control of TCM

In the cultivation of herbs, the quality of Chinese medicinal herbal products directly affects the quality of TCM. However, most of the cultivation, harvesting and processing management procedures of Chinese herbal medicinal materials are extensive. There is a lack of relevant technological innovations, the production process is not well scientifically based, the yield per unit area is low, the quality of the herbs is uneven, and the classification of species is not strictly enforced. There are no effective means to manage and resolve the problems of pesticide residues during pest control, and heavy metal residues occur through the process of planting, leading to the existence of unwanted chemicals in the herbs [34]. There are only a few quality control indicators for most of Chinese medicinal materials, and some of them have no quantitative indicators and even no qualitative indicators. Currently, the majority of the existing Chinese medicinal materials quality control specifications are not effectively to ensure the stable quality of Chinese medicinal materials, and the medicinal ingredients in these materials, which are the most direct causes for the unstable quality of TCM and the unevenness of its clinical efficacy [34].

TCM is more complex and comprehensive than Western medicine in terms of finished product quality, whether for a single drug prescription (prescriptions consisting of a single medicinal material) or a compounded Chinese medicine (prescriptions consisting of two or more medicinal materials). There is a lack of studies on material basis, active ingredients, and mechanism of action and no scientific and reasonable quality control indicators and methods [35]. Therefore, the uniformity, safety and effectiveness of inter-assay stability for TCM products cannot be guaranteed.

The implementation periods required by Chinese good clinical practice (GCP), good laboratory practice (GLP), GMP and other specifications are shorter than the international standards for pharmacology, toxicology, standardization, GMP construction, etc. The foundation is weak, various research data have not yet gained acceptance in international communities, and there is still a long way to go before TCM can harmonize with international standards [36]. Most TCM enterprises commonly experience the following problems: there is not enough application of advanced technology, and there is a large gap between TCM practices and international advanced technologies [37]. Although the implementation of a new version of the GMP forced out some non-standard enterprises, some Chinese medicine enterprises still use outdated hardware and facilities, including outdated technologies for extraction, concentration and purification of Chinese herbal medicines; therefore, some key parameters are unclear and cannot be effectively controlled. There is a large gap between China and developed countries in terms of the extraction process and production technology of natural medicine.

### Difficulty in identification of active principle from TCM composition

Regardless of specific national drug regulations there is an international consensus that all TCM drugs must meet stipulated high quality standards focusing on authentication, identification and chemical composition [38]. Chinese herbal medicine and TCM itself have complicated components that change constantly during the production of TCM, especially in the extraction process. Some components are not originally from Chinese herbal medicine itself but are transformed during the process of extraction and concentration, leading to difficulty in the identification of TCM components. In addition, TCM emphasizes mutual synergy, so it cannot simply use one or several indicators to fully represent the effect of TCM products. TCM consists of many chemical ingredients, and these ingredients play synergistic roles in human body [39], thus, the active medical ingredients are not clear. TCM adopts multi-target, multi-functional method to make overall adjustments and restore the human body to achieve the goal of treating both the manifestation of the disease and its cause. TCM is difficult to be clearly expressed by the chemical compositions in accordance with the requirements of chemical drugs, where the compositions are clearly expressed in chemical formulas.

At present, many enterprises and research institutions are committed to the pharmacology and composition of traditional Chinese medicine research. Advances in phytochemistry, high throughput screening, DNA sequencing, systems biology, and bioinformatics can reveal the chemical composition and molecular mechanisms of TCM [39–41]. In the TCM hospital Bad Kötzting, 171 TCM drugs underwent an analytical quality proof including thin layer as well as high performance liquid chromatography [38]. As from now mass spectroscopy will also be available as analytical tool. The findings are compiled and will be published one after another. The main issues of the analytical procedure in TCM drugs like authenticity, botanical nomenclature, variability of plant species, as well as medicinal parts processing are pointed out and possible ways to overcome them are sketched [38]. At present, the construction of chromatographic fingerprints plays an important role in the quality control of complex herbal medicines [42]. Many companies utilize advanced technologies to develop multi-component determination technics and chromatographic fingerprint analysis for qualitative and quantitative assessments. These assays will create a complete monitoring and evaluation system to ensure the efficacy, consistency, and inter-batch stability of the product. It is possible to clarify the role of the composition of TCM in the future.

### Limited research input into TCM and challenge for technological innovation

In recent years, Europe, the US, Japan, South Korea and many other developed countries and regions have adopted modern research methods and techniques to increase the development of traditional botanical drugs, the screening and confirmation of active ingredients, the establishment of international advanced quality standards and the development of new formulations [43]. Most domestic Chinese enterprises and research institutes make relatively less investment in the R&D of TCM. Insufficient R&D investment leads to a lack of competitiveness of the enterprise products. Chemical drugs and biological products enjoy obvious competitive advantages, seriously affecting the growth rate of the market share of TCM. TCM products feature more impurity or low purity and a lack of innovation in dosage forms; traditional dosage forms still hold a dominant position. The development and application of quick-acting, long-term, efficient and convenient emergency dosage forms and other new dosage forms are still in the beginning stage. The production and application of new pharmaceutical excipients are insufficient and have a large gap with the mainstream trend of international drugs, directly affecting the competitiveness of TCM in the international market [43].

## Opportunity analysis of internationalization of TCM

### Continuous improvement of attention to TCM by the international community

With the progress of society and changes in the human disease spectrum, the medical model has undergone tremendous changes; people are not simply seeking disease treatment but are focusing on the comprehensive management of disease prevention, treatment and health protection [44]. People are inclined to treat disease with natural medicine due to the obvious toxic and side effects of chemical drugs, and Chinese medicine is aligned with this development tendency [45]. In addition, many national governments have gradually accepted and attached importance to TCM and natural herbal medicine because of the high bio-pharmaceutical R&D costs, medical costs, long-term costs and other issues. The international community continues to accept natural medicine, and market demand will continue to grow. More than 90 countries and regions are introducing laws and regulations for the registration of Chinese herbal medicine [46].

TCM herbal drugs are increasingly used in many countries of the EU [47]. In Europe, it is very good to know that European Pharmacopeia (Ph Eur) is working on TCM herbal drug quality monographs. The European Directorate for the Quality of Medicines (EDQM) has established two groups of experts in pharmacognosy, who elaborate monographs on herbal drugs and herbal drug preparations. Since 2007 a special working group has been established with the elaboration of monographs on traditional Chinese medicinal plants and preparations [48]. Till now, a working program, existing of 75 monographs was established by the Commission of Ph Eur, out of which almost 50 new TCM herbal drug monographs have been implemented for the Ph Eur so far [49]. The standards put forward in these monographs not only define the quality of these products, but also eliminate dangerous counterfeit, substandard, adulterated and contaminated (traditional) herbal medicinal products [48].

With many TCM herbal drug monographs are implemented in the Ph Eur, this will be a significantly contribution to the acceptance of TCM worldwide [50]. All aspects relevant to the quality parameters have to be achieved in an adequate manner, requiring a broad range of analytical methods to be applied for new herbal drug monographs in the Ph Eur [47].

### Opportunity for economic globalization

First, due to the equivalent access for World Trade Organization (WTO) members, China’s admission to the WTO gave it more opportunities to participate in international exchanges and cooperation, to promote the wider spread of Chinese medicinal culture, and to recommend TCM products with minimal side effects and high efficiency in treating both symptoms and causes of disease, which will establish a good foundation for popularizing TCM in the international community [25]. Second, Chinese medicine is becoming more popular for treating and preventing many diseases, especially incurable diseases and chronic diseases, in many countries due to the poor efficacy and obvious side effects of Western medicine. Third, due to a decline in tariffs [51], many enterprises are more able and more willing to introduce foreign advanced technologies and equipment into China, which will speed up the production technology and accelerate the internationalization of traditional Chinese medicine.

### Chinese government policy support

In recent years, China has introduced a series of new policies and regulations to support the development of Chinese medicine, while increasing the support for international cooperation in Chinese medicine, indicating that the development of Chinese medicine has risen to a strategic governmental level. China attaches great importance to the inheritance and innovation of Chinese medicine, which will greatly promote the entry of Chinese medicine into the international market.

In addition, the implementation of the national strategy “One Belt and One Road” will create a new historical opportunity for Chinese medicine to move into the international community [52]. The development strategy of “One Belt and One Road”, utilizes the ancient “Silk Road” historical symbols, actively develops economic cooperation partnerships along with the countries with geographical proximity and cultural similarities to create great opportunities for the development of Chinese medicine industry [52]. With the “One Belt and One Road” strategy, we can promote the communication of TCM cultures. Through constantly TCM cultural exchange, we can bridge the gap between Eastern and Western medical systems to enhance the TCM internationalization.

At the same time, the advantages of TCM and the international market demand provide great possibilities for TCM under the “One Belt and One Road” strategy. TCM has the feature of both humanity and medical sciences, therefore, its great resource in the economy, cultural and health care, should be regarded as one of the best mediums of cultural exchanges and international cooperations between China and the countries along the “One Belt and One Road”. Moreover, “One Belt and One Road” strategy also provides the platform for inheritance and innovation of TCM, which will promote the further development of Chinese traditional medicine [53]. During this period, we can make full use of related resources and strengthen the international exchange and cooperation related to TCM. Cooperation and exchange include enhancing the policies and regulations for traditional medicine, improving herbal medicine quality standards and control indicators, and establishing administration regulations. There will be more exchanges between the Chinese government and international organizations, and the cooperation with foreign universities, research institutions, and hospitals as well, to co-establish academic and clinical research centres or groups to let more academic authority institutions accept and recognize TCM.

### Continuous recognition of the advantages of TCM

The unique role of TCM has been widely recognized by the international community in the process of severe acute respiratory syndrome (SARS) prevention and control and has been highly praised by the World Health Organization. Chinese medicine can effectively reduce the mortality rate of SARS patients and the sequelae in the process of treating the SARS virus, and the treatment costs are significantly reduced. The specific function and significance of Chinese herbal medicine in treating SARS have been recognized by WHO experts and have also helped reduce prejudice against the supposed inaccurate efficacy of TCM in the international community [54, 55].

A researcher at the China Academy of Chinese Medical Science, Tu Youyou, was awarded the 2015 Nobel Prize in Physiology or Medicine for the development of artemisinin. Tu Youyou made an exploratory investigation of TCM and found that the components extracted from the plant *Artemisia annua* are effective in the treatment of malaria. By referring to ancient books and many studies, Tu Youyou found the best extraction method for artemisinin. Artemisinin is the invention not only of Tu Youyou but also of China and is an achievement of TCM heritage and innovation, which deserve popularization. This important invention created a sensation in the world [14].

## Threat analysis of the internationalization of TCM

### Threats posed by differences between Chinese and Western cultures

Due to differences between Chinese and Western cultures, China’s TCM theory cannot be accepted by the international community [56]. TCM theories advocate “discontinuing medication when the patient is cured”, oppose the long-term medication. Long-term medication or even increase of dose to several times of standard dose may inevitably lead to side effect [57]. This also exposes the differences between Chinese and Western cultures. Chinese medicine emphasizes the principle of syndrome differentiation. However, from Western medicine perspective the concept of TCM is hardly understood and accepted, therefore TCM is not well positioned and accepted [5].

### Dual threats in Chinese and foreign markets

The officially entering of TCM to Europe Union (EU) market is at a slow rate, and there are only four products produced by Chinese enterprises registered in the EU; no real TCM is sold in the US; and the TCM industry is facing enormous challenges. China’s botanical drugs have only a small share, less than 5%, of the international botanical market, 2% in the United Kingdom and the US, and 0.2% in Germany [58]. As natural medicine has been recognized by the international community, TCM and natural medicine have become the target of many large multinational companies. The international herbal medicine market is highly competitive. At present, Japan, South Korea and other countries are competitive with China in the international herbal medicine market and pose a great threat to China’s exports.

In addition, a large number of foreign TCM companies based in Japan, South Korea and India have impacted the TCM market in China. China is the world’s largest TCM market, and its demand for natural medicine is also very large. In recent years, due to the continuous opening of China’s pharmaceutical market, many large-scale foreign pharmaceutical enterprises have entered the Chinese market, and approximately 40 types of natural medicine manufactured in more than 10 countries and regions are successfully registered and listed in China [59].

### Limitation of technical trade barriers

The practice standards of China’s TCM industry remain in the progress of standardization. After entering the WTO, China had to align its standards with international standards. However, many countries and regions in the world, especially the Western developed countries, restrict foreign products entering their territory through various administrative measures and requirements, high-tech trade barriers and the “green trade barriers” threshold, including measures for medication safety and protection to strengthen the supervision of imported drugs; develop or improve relevant technical requirements, such as quality standards for heavy metal residues, pesticide residues, aflatoxin and others; standardize the technology of plant extracts and environmental standards; and so on [42].

### Threats to intellectual property related to TCM

Chinese medicine has a 5000-year history in China, and in a traditional comprehensive theoretical system, the related intellectual property should belong to China. However, the implementation of patent protection and technical protection of the novel TCM drugs in China began late, and there is a lack of study of protection of TCM intellectual property rights; therefore, most Chinese medicine enterprises lack experience in patent applications in foreign countries and are not ready to protect their intellectual property. Most TCM can easily be imitated or limited by patents in foreign countries; thus, many valuable Chinese medicine product technologies and knowledge are under threat of advanced applications for patents by foreign enterprises. The developed countries, which have rich experience in drug patents, can not only obtain for TCM intellectual property rights but also compete for other resources related to Chinese medicine by using intellectual property advantages.

## Summary and perspective

With the SWOT analysis of the internal and external environments, we can observe the advantages, disadvantages, opportunities and threats of TCM during the internationalization process (Table 2). Overall, the advantages outweigh the disadvantages, and the opportunities outweigh the threats.

**Table 2:** SWOT analysis in traditional Chinese medicine internationalization

Subjects	Criteria description
Strength	TCM in theory and in treating diseases International demand for TCM Natural medicine resources The scale and power of TCM enterprises
Weakness	Cultural diversity Quality control of TCM Difficulty in identification of active principle from TCM composition Limited research input into TCM and challenge for technological innovation
Opportunity	Continuous improvement of attention to TCM by the international community Opportunity for economic globalization Chinese government policy support Continuous recognition of the advantages of TCM
Threat	Differences between Chinese and Western cultures Dual risks in Chinese and foreign markets Limitation of technical trade barriers Challenges in intellectual property related to TCM

To deal with these disadvantages and threats, we can provide some recommendations. First, we can strengthen the international communication on Chinese culture. We should make full use of related resources at home and strengthen the international exchange and cooperation related to TCM, thereby promoting the development of TCM culture. We should strengthen the exchange between the Chinese government and the leadership of international organizations; strengthen the cooperation with foreign institutions of higher educations and hospitals and co-establish clinical research centers or groups to make more academic authority institutions accept and recognize TCM.

Second, we can increase the input into TCM research. To align the foreign regulations concerning natural medicines, TCM can start with each individual medicinal materials. Enterprises can take some multi-herbal formula of TCM with specific curative effects, and take the effective part as an “effective body”, then control the composition and contents of the “effective body”, and gradually reach the purposes of “safe, effective, controllable, and stable”. The “effective body” in individual medicinal material can also be analyzed and summarized with the “assistant and guide” theory of TCM. We should explore and investigate the traditional formulas and carry out secondary development with modern innovative technologies to develop and produce new products.

Third, we can enhance the standardization and modernization of TCM quality. We should issue TCM product quality standards that are applicable to both China and the world as soon as possible and establish strict management of planting and production, to ensure that the consistency of TCM quality can be controlled. We should regulate each step of the TCM industrial process, apply high and new technologies, use advanced equipment and adopt strict standards to transform the TCM production and reach the goals of modernizing the technologies for the extraction, formulation, and quality control of TCM based on related conventional theories.

Finally, we must carry out intellectual property protection training among the practitioners of the TCM industry and enhance the awareness of the whole industry regarding the protection of intellectual property. We must learn the domestic and international regulations on the protection of intellectual property. We may also achieve effective protection through the core technologies, trademarks, essence and inventions in TCM intellectual property.

In the process of the internationalization of TCM, we must emphasize the advantages to avoid the disadvantages, seize the opportunity to overcome the threats, carry out specific internationalization processes, avoid detours and promote a rapid internationalization process.

## Abbreviations

TCM: traditional Chinese medicine
DM: diabetes mellitus
R&D: research and development
US: the United States
GMP: good manufacturing practice
GCP: good clinical practice
GLP: good laboratory practice
Ph Eur: European Pharmacopoeia
EDQM: European Directorate for the Quality of Medicines
WTO: World Trade Organization
SARS: severe acute respiratory syndrome
EU: Europe Union
